# Are there predilection sites for intracranial meningioma? A population-based atlas

**DOI:** 10.1007/s10143-021-01652-9

**Published:** 2021-10-21

**Authors:** Sayied Abdol Mohieb Hosainey, David Bouget, Ingerid Reinertsen, Lisa Millgård Sagberg, Sverre Helge Torp, Asgeir Store Jakola, Ole Solheim

**Affiliations:** 1 Department of Neurosurgery, Bristol Royal Hospital for Children, Bristol, BS2 8BJ UK; 2 Department of Health Research, SINTEF Technology and Society, Trondheim, Norway; 3 Department of Circulation and Medical Imaging, Norwegian University of Science and Technology, Trondheim, Norway; 4 Department of Neurosurgery, St. Olavs Hospital, Trondheim, Norway; 5 Department of Neuromedicine and Movement Science, Norwegian University of Science and Technology, Trondheim, Norway; 6 Department of Laboratory Medicine, Children and Women’s Health, Faculty of Medicine, Norwegian University of Science and Technology, Trondheim, Norway; 7 Department of Pathology and Medical Genetics, St. Olavs Hospital, Trondheim, Norway; 8 Department of Neurosurgery, Sahlgrenska University Hospital, Gothenburg, Sweden; 9 Institute of Neuroscience and Physiology, Department of Clinical Neuroscience, University of Gothenburg, Sahlgrenska Academy, Gothenburg, Sweden

**Keywords:** Brain tumor, Meningioma, Tumor location, Predilection site, Tumor atlas

## Abstract

Meningioma is the most common benign intracranial tumor and is believed to arise from arachnoid cap cells of arachnoid granulations. We sought to develop a population-based atlas from pre-treatment MRIs to explore the distribution of intracranial meningiomas and to explore risk factors for development of intracranial meningiomas in different locations. All adults (≥ 18 years old) diagnosed with intracranial meningiomas and referred to the department of neurosurgery from a defined catchment region between 2006 and 2015 were eligible for inclusion. Pre-treatment T1 contrast-enhanced MRI-weighted brain scans were used for semi-automated tumor segmentation to develop the meningioma atlas. Patient variables used in the statistical analyses included age, gender, tumor locations, WHO grade and tumor volume. A total of 602 patients with intracranial meningiomas were identified for the development of the brain tumor atlas from a wide and defined catchment region. The spatial distribution of meningioma within the brain is not uniform, and there were more tumors in the frontal region, especially parasagittally, along the anterior part of the falx, and on the skull base of the frontal and middle cranial fossa. More than 2/3 meningioma patients were females (*p* < 0.001) who also were more likely to have multiple meningiomas (*p* < 0.01), while men more often have supratentorial meningiomas (*p* < 0.01). Tumor location was not associated with age or WHO grade. The distribution of meningioma exhibits an anterior to posterior gradient in the brain. Distribution of meningiomas in the general population is not dependent on histopathological WHO grade, but may be gender-related.

## Introduction

Meningiomas account for approximately 1/3rd of all CNS tumors with an age-adjusted incidence rate of 8.58 per 100,000 [30]. According to the World Health Organization classification of CNS tumors, meningiomas are classified into grades I, II, and III based on histopathological features such as cellularity, cell architecture, necrosis, and invasiveness of the tumor [22]. Meningiomas are believed to arise from arachnoid cap cells in the arachnoid granulations due to the histological similarities between meningioma cells and arachnoid villi cells [36, 45]. The arachnoid granulations are unevenly distributed throughout the brain and across the venous system [3]. However, it is not known if the distribution of arachnoid granulations can be linked to the anatomical distribution of meningioma.

Meningiomas are clinically and radiologically often classified according to their location and several different anatomical classifications are in use. However, distribution of meningiomas has not been systematically studied from a population-based angle, and it is therefore not known whether meningiomas have special predilection sites with respect to different patient characteristics. Even so, different outcomes have been associated to different tumor locations [4], but relation between for instance WHO grade and tumor location, age and tumor location, sex and tumor location, or tumor volumes at diagnoses and tumor location has not been coherently investigated.

In the present population-based study, we sought to explore the anatomical distribution of meningioma with a map-based topographical approach to assess if there are predilection sites of meningioma and if so, explore different factors that are associated with tumor location.

## Methods and materials

### Study population

Adult patients, 18 years or older, with radiologically or histopathologically confirmed meningioma referred to the Department of Neurosurgery, St. Olavs University Hospital, from 2006 through 2015 were eligible for inclusion. Except for two patients who had emigrated from Norway, no patients were lost to follow-up. St. Olavs University Hospital serves exclusively as the neurosurgical center in a geographically defined catchment region with approximately 750,000 inhabitants.

The diagnostic ICD-10 (International Classification of Diseases) codes D32 and R90 were used to screen potential eligibles for inclusion. In patients who had undergone surgery, final histological diagnosis of meningioma was verified by review of pathology reports. Meningiomas were classified based on the 2007 WHO classification [21]. For patients who did not require early treatment, but were followed as outpatients with a wait-and-scan approach, either by the neurosurgery department or at a local hospital, inclusion was based on a confirmed radiological diagnosis of meningioma, typically showing homogenous contrast enhancement and “dural tails” on T1-weighted MR images [7, 39].

**Fig. 1:**
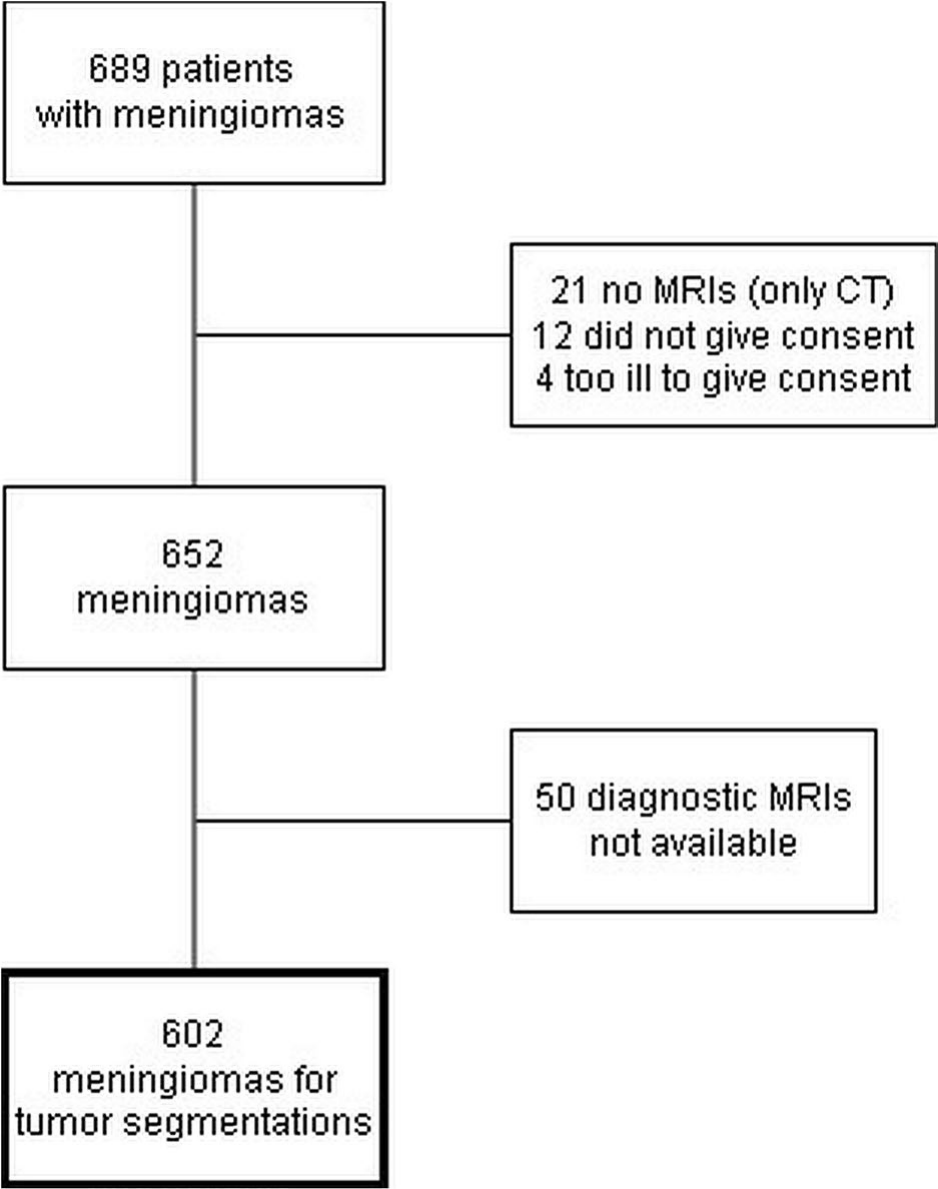
Flowchart demonstrating number of patients with meningiomas included in developing the brain tumor atlas

Meningioma patients who previously had signed written informed consents to participation in a prospective, local brain tumor registry were all included. Additionally, patients who were dead (and who earlier had not declined participation in the registry) were also included. Moreover, patients who were not included in the tumor registry (mainly patients who had not undergone surgery but were followed as outpatients) received a letter with information about the study and were given an opportunity to withdraw from the study with an active decline by returning a prepaid envelope. Exclusion criteria were patients with active decline from study participation, patients where diagnostic MRIs were missing, patients who had undergone head CTs only (due to contraindications such as to pacemakers, pregnancy, and claustrophobia), and patients who had emigrated from Norway. Patients without earlier consent in the prospective registry who were not able to sign informed consents due to severe functional or cognitive deficits including severe psychiatric disorders were not asked to participate in the study. A flow chart of the inclusion/exclusion process is shown in Fig. 1.

### Data collection

Demographic data was retrieved from our prospectively maintained surgical tumor registry at St. Olavs University Hospital, and the data were reviewed retrospectively. For non-operated patients and patients who were not included in the registry, retrospective reviews of electronical medical records (from St. Olavs University Hospital and seven local hospitals in our catchment region) were carried out. This included patients who were seen as outpatients only and/or those where referrals had been managed by letters giving advice on follow-up locally after diagnostic MRI scans were reviewed by the neurosurgical department.

The following data were retrieved: age, gender, WHO grade (I, II, or III) as verified by histopathology, radiological diagnosis, gross tumor location (supratentorial/infratentorial/both supra- and infratentorial tumor location), specific tumor location adapted from Youmans Neurological Surgery [44] with slight modification (Table 1), and tumor multiplicity (defined as tumors in separate locations in the same patient). Diagnostic/pre-treatment MRI scans were retrieved for segmentation of tumor volume (cm^3^/ml). To determine whether the distribution of meningiomas was associated with menopausal status, female sex was dichotomized to < and ≥ 50 years old.

**Table 1:** Patients and meningioma characteristics

	*N*	%
Age—median (range)	62.4 (18.2–92.7)	-
Sex		
Male	170	28.2
Female	432	71.8
Females < 50 years	83	19.2
Location		
Supratentorial	508	84.4
Infratentorial	93	15.4
Supra- and infratentorial	1	0.2
Right/left/midline	277/252/73	46.0/41.9/12.1
Multiple locations	60	10.0
Tumor location (specific)^a^		
Convexity	149	24.7
Parasagittal	99	16.4
Falx	46	7.6
Tentorium	25	4.2
Olfactory groove	47	7.8
Sphenoid wing and clinoid	96	15.9
Tuberculum sellae	23	3.8
Cavernous sinus	13	2.2
Cerebellum convexity	31	5.2
CP angle	36	6.0
Clival and petroclival	15	2.5
Foramen magnum	6	1.0
Optic n. and orbital	13	2.2
Intraventricular	1	0.2
Pure intraosseus	2	0.3
Tumor volume (range)^b^	6.2 (0.1–168.0)^d^	
Surgical treatment	18.8 (0.7–168.0)	-
Conservative management	2.9 (0.1–74.1)	-
WHO grade (I/II/III)^c^	198/61/2	75.8/23.4/0.8
Total	602	100

^a^Adapted from Youmans Neurological surgery with slight modifications

^b^Pre-registration median volume (cm^3^/ml)

^c^This constitutes only those who had surgery (*n* total = 261) with pathological reports. Non-surgical patients (*n* total = 341) had radiologically confirmed meningioma verified by neuroradiologists

^d^Overall tumor volume of all meningiomas

### Development of brain tumor atlas

Preoperative contrast-enhanced T1-weighted MRI sequences acquired on 1.5- or 3-T scanners were retrospectively obtained from the hospital’s radiology database for patients who had undergone surgery. The first MRI sequence with radiologically confirmed meningioma was retrieved from the radiology database for patients that were followed up conservatively.

In each case, DICOM (Digital Imaging and Communications in Medicine) format images were anonymized and imported to 3D Slicer software version 4.5.0 (www.slicer.org) for semi-automatic tumor segmentation, and the T1-weighted gadolinium-enhanced MRI sequence volumes (axial, sagittal, or coronal axis view) with maximum number of image-slices per volume were uploaded to the workspace. For both solitary and multiple meningiomas, the entire pathological contrast-enhanced region of the tumor(s), including the “dural tail,” was delineated and segmented with the competitive region-based segmentation “GrowCutEffect” module of the software [6]. Subsequently, if regions outside the tumor borders were automatically segmented by the software, the non-contrast-enhanced regions were manually adjusted to produce the final segmented tumor.

After completion of tumor segmentations, all the MR images and corresponding segmentations were spatially aligned with a pre-defined brain template from the Montreal Neurological Institute (MNI—ICBM-152 average brain), which is used as standardized reference frame. Specifically, the ICBM2009a Nonlinear Symmetric was utilized, which is constructed from 152 healthy adult volunteers [9]. In particular, by utilizing the Advanced Normalization Tools (ANTs) framework [2] the segmented images were spatially aligned with the average brain by intensity-based registration. Initially, each of the images were pre-processed and skull-stripped to exclude non-brain structures using a neural network model pre-trained with over 300 samples. The architecture used is a regular 3D U-Net [38], and the implementation has been done using Keras and Tensorflow. The symmetric diffeomorphic technique from ANTs (named SyN) was used to register each of the image volumes to the template. Then, the resulting transformation was applied to the individual tumor segmentations in order to merge all the tumors into their common space, yielding the final atlas of meningiomas. Tumor volumes were registered onto the “common brain” to produce the final meningioma atlas with a strong positive and significant correlation between the pre- and post-registration tumor volumes (*R*^2^ adjusted = 0.97, *p* < 0.001).

### Statistics

Standard descriptive and quantitative statistical analyses were conducted with JMP 9.0 software (SAS Inc.). Chi-square (*X*^2^) and Fisher’s exact test were used for comparison between categorical variables as appropriate. Analysis of variance (ANOVA) and Student’s *t* test were used for continuous variables. Statistical significance was set at *p* < 0.05. Risk stratification of different variables was conducted where applicable.

## Results

### Patient population

**Fig. 2:**
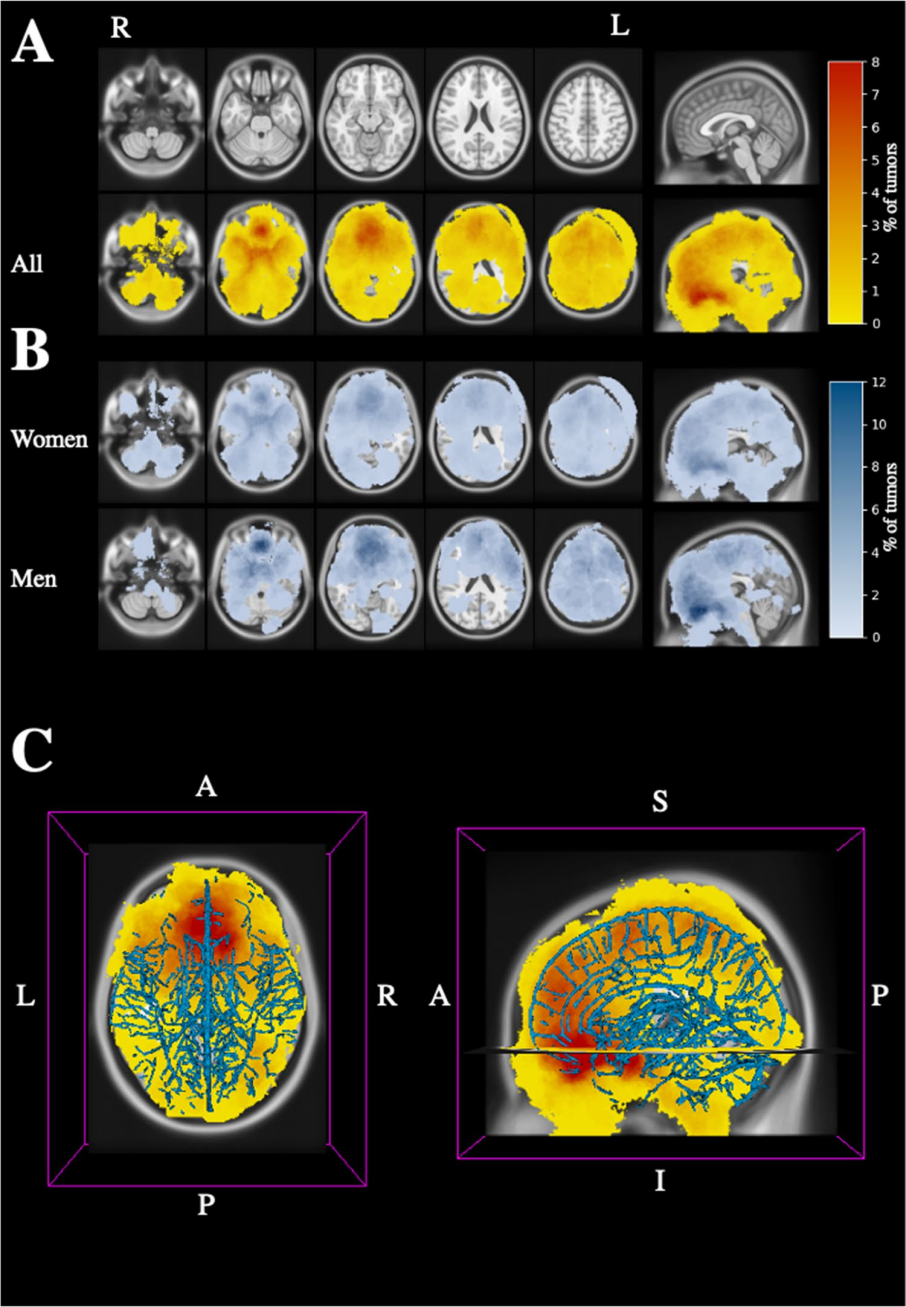
Distribution of intracranial meningiomas. Percentages of tumors are shown of all patients in yellow-orange-red color (**A**) with baseline T1-weighted MRI of normal brain in top row and in blue color for women and men in (**B**). Scalar bars for each volume is shown on the right. Meningioma locations shown in relation to venous drainage system is illustrated in (**C**). The image plane coordinates are depicted as *z* coordinate and *x* coordinate for the axial images and sagittal planes in standard MNI space, respectively

**Fig. 3:**
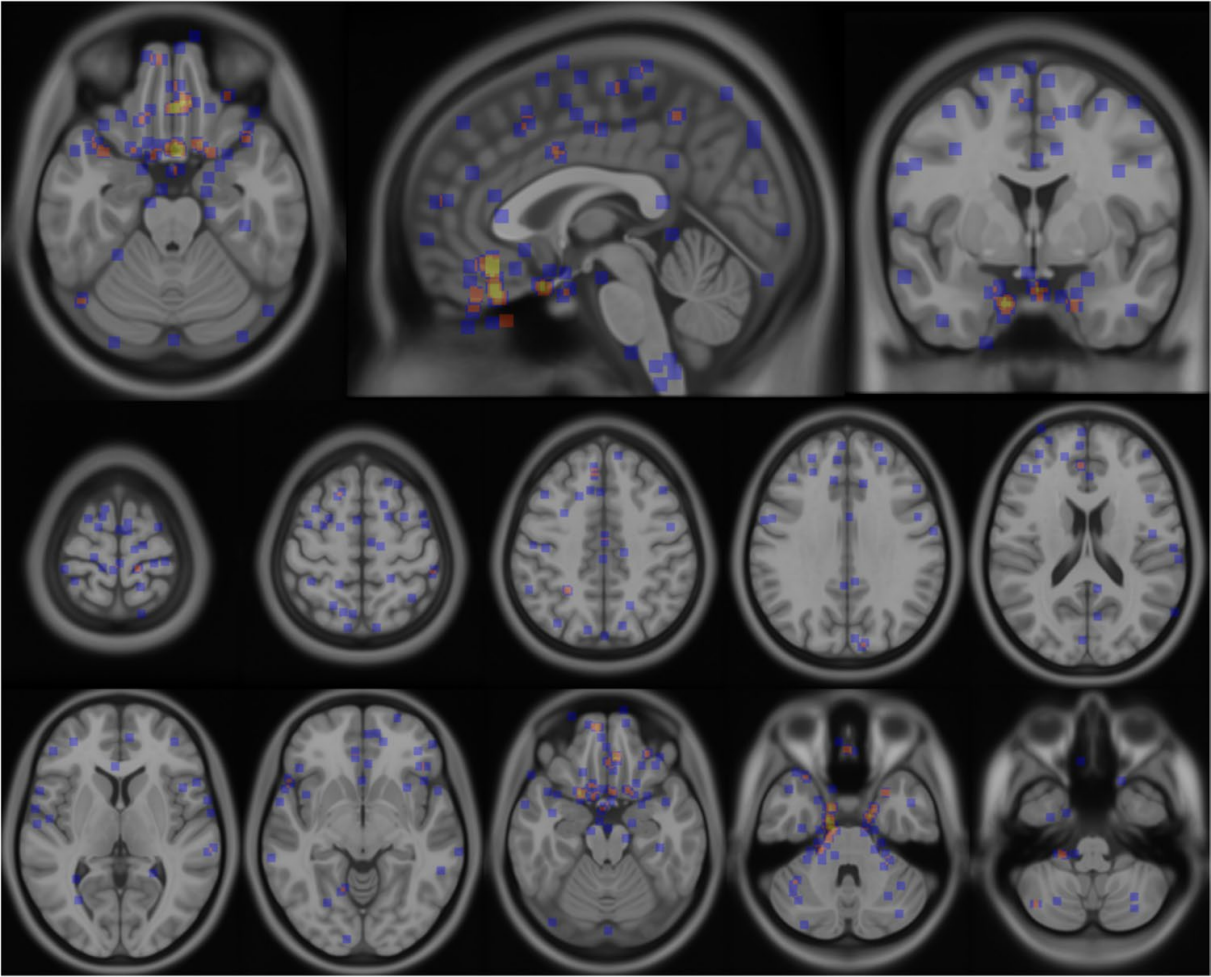
Meningioma distribution map based on central point of tumor mass (centroids). Orange/yellow color of centroids indicates locations with higher frequencies. Dark blue color of centroids depicts areas with lower number of meningiomas

A total of 689 adult patients with meningiomas were identified. After the exclusion process (Fig. 1), the images of 602 patients were included in the final analyses and registration onto common brain to produce the brain tumor atlas (Figs. 2 and 3). During the study period, 261 underwent surgery, while 341 were followed as outpatients with conservative management.

### Meningioma distributions

Meningiomas were located supratentorially in 508 patients (84.4%), infratentorially in 93 patients (15.4%), while one patient (0.2%) had both a supra- and infratentorially located meningioma. There were 277 meningiomas (46%) located in the right hemisphere, 252 were left sided (41.9%), while 73 meningiomas (12.1%) were located strictly in the midline. A total of 60 patients (10%) had multiple meningiomas (Table 1). With respect to specific tumor locations, the largest entity was convexity tumors (24.7%). In contrast, only one patient (0.2%) had an intraventricular meningioma (Table 1). As illustrated in Figs. 2 and 3, meningiomas are more frequently located in the anterior part of the brain along the convexity, falx, and parasagittally as depicted by the heat map. There are also more meningiomas on the anterior skull base region with gradual decline towards the posterior cranial vault and foramen magnum region. The distribution of meningioma is compared to the distribution of larger cerebral veins in Fig. 2c.

### Age

Median overall age for all patients was 62 years (range 18–92), where the median age of patients who underwent surgery or were followed conservatively was 58 years (range 18–86) and 64 years (range 18–92), respectively (Table 1). There were no significant associations between age and supratentorial, infratentorial, or both tumor locations (*p* = 0.59), right, left, or midline tumor location (*p* = 0.30), multiple tumor locations (*p* = 0.40), or specific tumor locations (*p* = 0.60).

### Gender

There were 170 (28.2%) male patients and 432 (71.8%) females of which 83 females were < 50 years old (Table 1). Overall, more women had meningiomas than men (*p* < 0.001) (Fig. 2). Females were also more likely to have multiple meningiomas in univariate analysis (OR 2.8, CI [1.3–6.0], *p* < 0.01), while men had higher risk of having meningiomas in the supratentorial compartment (OR 2.5, CI [1.4–4.5], *p* < 0.01). There were no significant associations between gender and right, left, or midline tumor locations (*p* = 0.28), or specific tumor locations (*p* = 0.06).

There were no significant differences in distributions of meningiomas between females < and ≥ 50 years old in supratentorial, infratentorial, or both locations (*p* = 0.99), multiple locations (*p* = 0.43), right, left, or midline locations (*p* = 0.36) or specific locations (*p* = 0.07). Females < 50 years old had significantly higher risk of having multiple meningiomas compared to males (OR 3.5, CI [1.4–9.2], *p* < 0.01), but not to females ≥ 50 years. No statistical significance was detected with respect to females < 50 years old and males in right, left, or midline locations (*p* = 0.36) or specific locations (*p* = 0.07).

### WHO grade

Among patients who underwent surgery, there were 198 WHO grade I meningiomas, 61 WHO grade II meningiomas, and 2 WHO grade III meningiomas (Table 1). There were no significant associations between WHO grades I vs. II/III and supratentorial or infratentorial locations (*p* = 0.20), right, left, or midline location (*p* = 0.06), multiple meningiomas (*p* = 0.29), or specific tumor locations (*p* = 0.34).

### Tumor volume

Median overall tumor volume was 6.2 cm^3^ (range 0.1–168) (Table 1). At diagnosis, tumor volumes were significantly larger in the supratentorial compartment compared to infratentorially (median 6.4 cm^3^ vs. 3.9 cm^3^ respectively, *p* < 0.001), but were not significantly associated with right, left, or midline tumor location (*p* = 0.44), multiple meningiomas (*p* = 0.36), or specific tumor locations (*p* = 0.10) (Table 2). Stratified analysis of tumor volume with respect to age, gender, and females < or ≥ 50 years old revealed that males have significantly larger tumor volumes than females overall (*p* < 0.01). There were no statistically significant associations between tumor volume and age (*p* = 0.62) or associations between tumor volumes in females < or ≥ 50 years old (*p* = 0.07).

**Table 2:** Relative associations of different variables with tumor locations^a^

	Age	Sex (*N*, %)		WHO grade (*N*, %)			Tumor volume (cm^3^)
	Median (range)	Female	Male	I	II	III	Median (range)
Supratentorial Infratentorial	62.4 (18.2–92.7) 62.4 (28.5–86.0)	352 (81.5) 80^b^ (18.5)	156 (91.8) 14 (8.2)	450 (83.5) 89^b^ (16.5)	57 (93.4) 4 (6.6)	1 (50) 1 (50)	6.4 (0.1–168.0) 3.9 (0.1–112.0)
Right Left Midline	63.8 (21.6–89.3) 61.0 (18.2–92.7) 64.0 (18.2–85.1)	205 (47.4) 180 (41.7) 47 (10.9)	72 (42.4) 72 (42.4) 26 (15.2)	240 (44.5) 231 (42.9) 68 (12.6)	35 (57.4) 21 (34.4) 5 (8.2)	2 (100)	6.6 (0.1–168.0) 5.5 (0.1–165.4) 6.3 (0.5–83.3)
Multiple locations	61.3 (18.9–88.7)	52 (12.0)	8 (4.7)	53 (9.8)	6 (9.8)	1 (50)	7.9 (0.5–165.4)

^a^Percentages (%) calculated vertically

^b^Including one patient (0.2%) with both supratentorial and infratentorial tumor location

## Discussion

In this population-based study, we developed a volumetric brain tumor atlas of all intracranial meningiomas from a total of 602 patients referred from a well-defined geographical catchment region. We also explored different patient characteristics to determine whether there are risk factors for developing meningiomas related to various intracranial sites. The segmented tumor volumes were registered and merged by aligning them onto a standardized “common brain” to develop the atlas of meningiomas. With respect to distribution of intracranial tumors, meningiomas were often located in the frontal convexity region and parasagittally, along the falx anteriorly and on the skull base region of the frontal and middle cranial fossa, with gradual decline in frequencies posteriorly towards the posterior fossa and foramen magnum region. Thus, although clinically useful, the common neuroanatomical classifications of meningiomas into specific and traditional anatomical locations may be seen as arbitrary and does not directly reflect predilection sites.

The meninx, which is the primordium for the meninges, the skull, and the scalp, is formed by differentiation of a mesoderm- and neural crest–derived layer that stem from mesenchymal cells completely encasing the brain and spinal cord during embryological development [29]. Meningiomas are believed to arise from the arachnoid cap cells of the arachnoid granulations, which protrude into the venous sinuses and form “portals” essential for absorption of cerebrospinal fluid (CSF). Although arachnoid granulations are more numerous along the intracranial venous sinuses and in particular along the parasagittal plane adjacent to the superior sagittal sinus [20], our atlas of meningiomas also demonstrate that the distribution of meningiomas does not follow the distribution of major veins or venous sinuses. In a study by Hirayama et al. of patients with meningiomas, they speculated that anatomical locations with high proportions of arachnoid cells are more frequently prone to harbor meningeal neoplasms [13]. However, their study concentrated on lesions on one side of the brain only in selected patients, whereas our population-based study included all patients with meningiomas who underwent surgery and/or were treated conservatively. Imaging studies of arachnoid granulations located along the main intracranial venous sinuses have been reported [10, 19], but distribution maps of arachnoid granulations within the meningeal layers covering the entire brain exploring predilection sites for development of meningiomas are scarce. Nevertheless, there is no known anterior- to posterior gradient of arachnoid granulations in the brain [10], suggesting that there is not a perfect association between the density of arachnoid granulations and meningiomas. Also, CSF efflux along the parasagittal dura is greater in the middle to posterior segments of the superior sagittal sinus [37], which could indicate more numerous arachnoid granulations in these regions, rather than anteriorly. However, more meningiomas are located anteriorly in the brain according to our study. Interestingly, even though the falx cerebri is shorter/narrower in its anterior portion while the posterior portion is broader and attaches to the tentorium cerebelli [35], meningiomas were most abundant along the convexity and anterior cranial vault compared with the posterior region. Molecular stem cell marker positive cells associated with meningiomas have been reported in which some histological tumor types are more frequently located in non-skull base regions [1, 18] suggesting that potential stem cells may be widely spread among different regions of the brain. In the early embryonic and early postnatal development, a prostaglandin D2 synthase (PGDS)–positive meningeal precursor has been indicated to play a role in meningioma formation. This also accounts for the different meningioma subtypes when the biallelic NF2 gene is inactivated [17]. Also, PDGS-positive meningeal cells have been identified as a common precursor to both the dural border cells and arachnoid border cells [46]. Vascular supply might also play a role in predilection of tumor and tumor growth as meningiomas. The vascular construction forms a more complex network in the cranial base than over the convexity. The dural territories often have overlapping vascularization from several sources such as in the parasellar dura, tentorium, and falx [24]. Furthermore, dural anatomy such as the single-layered dura of the medial wall of the cavernous sinus [47] may also play a role in growth of meningiomas in different intracranial regions.

The locations of meningiomas in the present study were comparatively similar overall to published series on both conservatively managed and/or surgically treated meningiomas [15, 25, 28, 31, 34], depending on how tumor locations are categorized. Also, meningiomas were more often seen in women (male to female ratio 1:2.5), a finding consistent with a population-based cancer registry report [14], and most meningiomas were convexity tumors representing approximately one-quarter of all tumors, in line with previous reports [15, 16, 28, 31, 34]. We did not find any significant associations between age and tumor locations, in contrast to a study by Sun et al. where frontal and occipital structures were more frequently associated with older patients, males, and high-grade meningiomas [43]. However, their study was restricted to surgical cases only. Some reports have dichotomized meningioma locations to skull base and non-skull base regions where the ratio of females was significantly higher in the skull base region [25, 26, 41, 42]. In comparison, we found that men are more likely to have meningiomas located supratentorially compared to females. Thus, hormonal factors may play a role in tumor distribution. Females younger than 50 years old were also more likely to have multiple meningiomas than men in our study, but not compared to females ≥ 50 years old. Meningiomas can be associated with progesterone and estrogen receptor activity [33], and hormone replacement therapy (HRT) has been associated with higher risk of meningiomas in females 26–55 years of age compared to controls [5]. However, in a large nationwide population-based study of women 15–57 years old, pregnancy was shown to rather decrease the risk of meningioma during pregnancy and during follow-up after childbirth compared to nulliparous women [32]. Nevertheless, no specific predilection sites were detected for meningiomas in females less than 50 years old in our study.

In contrast to some studies where high-grade meningiomas more frequently were found over the convexity [11, 23, 34, 43] and non-skull base regions [18, 40], we found no such association between WHO grade and tumor locations. In our population-based selection, the overall median tumor volume was 6.1 cm^3^ (mean 16.8 cm^3^), which is somewhat larger than in reports of incidentally discovered and surgically treated meningiomas [8, 12, 23, 27]. However, these studies are restricted to certain tumor locations only and warrant further studies as comparison to our study was difficult due to lack of reports in the literature where similar risk factors are explored. Meningiomas were significantly larger in the supratentorial compartment and in males in our study, but we did not find any significant association between tumor volume and tumor locations. In a study by Magill et al. of 1113 meningioma patients, male patients with tumors > 3 cm were identified as high-risk group for WHO grade II meningioma [23]. However, their study of surgically resected cases only did not account for volume with respect to tumor location and volumetric analysis of tumor was not performed (only tumor diameter was used as proxy), whereas our study was comprised of volumetric tumor segmentation of all included patients with meningiomas from a large well-defined geographical catchment region.

### Strengths and limitations

The centralized neurosurgical tertiary health care center at St. Olavs University Hospital has a population-based referral of patients from a wide catchment area. To the best of our knowledge, our study is the first to report an intracranial atlas of meningioma distribution from a well-defined geographical region, thus reducing risk of referral bias. This centralization of neurosurgical services minimizes possible confounding effects of differences in access to health care services. Hence, we have avoided the selection bias inherently present in large multicenter studies, as there is only one unit performing neurosurgical procedures for meningiomas. By adhering to the WHO classification of tumors, we have confirmed histological verification of meningiomas for those who underwent surgical intervention, thus improving the external validity of our results. Whereas some similar reports have studied meningioma distributions in selected patients and/or tumor locations, our study is population-based including both patients who did and did not undergo surgical intervention for meningioma in which patient characteristics were explored as risk factors for predilection sites of intracranial meningiomas. All tumor segmentations was performed manually and assessed in a 3D map-based approach, thereby minimizing the risk of classification bias. Finally, as only two patients emigrated abroad, we have effectively no loss of follow-up of patients in our study.

Possible limitations of the study include a potential discrepancy between histologically and radiologically diagnosis of meningiomas where the former is established as the gold standard. Even though it is commonplace and routine practice within the Norwegian Health Care system to refer most patients with meningiomas to the neurosurgical treatment center for treatment or recommendations concerning follow-up, there is still a possibility of underrepresentation of patients with tumors of small sizes and/or the oldest age group harboring meningiomas who might have not have been referred, but rather managed locally (depending on their comorbidities/circumstances). We also did not account for patients with respect to pregnancy, as this might influence the occurrence of meningiomas due to hormonal changes during pregnancy and after childbirth.

## Conclusion

In this large population-based study, we developed an atlas of intracranial meningioma distribution. As illustrated, meningiomas are more often located in the frontal region in the convexity and parasagittally, along the falx anteriorly, and on the skull base of the frontal and middle cranial fossa, with gradual decline in frequency posteriorly towards the posterior fossa and foramen magnum region. Women had higher risks of having multiple meningiomas, while supratentorial location is more common in men. Age and histopathological WHO grade were not linked to tumor location.

## Data Availability

Data may be given upon reasonable request
